# Improving the poor diagnostic accuracy of the ECG at detecting prognostically significant left ventricular hypertrophy in hypertensive patients

**DOI:** 10.1186/1532-429X-17-S1-P324

**Published:** 2015-02-03

**Authors:** Jonathan C Rodrigues, Bethannie McIntyre, Amardeep Ghosh Dastidar, Amy E Burchell, Laura E Ratcliffe, Emma C Hart, Julian F Paton, Chiara Bucciarelli-Ducci, Mark Hamilton, Angus K Nightingale, Nathan E Manghat

**Affiliations:** 1 CMR Unit, NIHR Cardiovascular Biomedical Research Unit, Bristol Heart Institute, Bristol, UK; 2 School of Physiology and Pharmacology, The University of Bristol, Bristol, UK; 3 Foundation School, Severn Postgraduate Deanery, Bristol, UK; 4 Cardionomics Research Group, Bristol Heart Institute, Bristol, UK

## Background

Normalised left ventricular mass (LVM) is a powerful prognostic tool. Traditionally, normalising has been achieved by indexing LVM to body surface area (BSA). A recent ‘Multiethnic Study of Atherosclerosis' (MESA) sub-study demonstrated indexing LVM to height^1.7^ is more sensitive at identifying left ventricular hypertrophy (LVH) associated with cardiovascular events and all-cause death. We evaluated the ability of the ECG, an universal investigation in patients with hypertension, to detect LVH defined traditionally by LVM/BSA and by the prognostically more important LVM/height^1.7^ method using CMR (non-invasive gold-standard for LVM).

## Methods

111 consecutive patients (mean age: 52.1±14.4 years, 51.4% male) from our tertiary hypertension clinic who underwent CMR were included. LVM was estimated using established CMR methods. Papillary muscles were included in LVM, using blood thresholding contouring software. LVH was defined as >95% confidence interval of normal references values for LVM/BSA and for LVM/height^1.7^ respectively. A contemporaneous 12-lead ECG was assessed, by a clinician blinded to CMR data, for the following LVH criteria: Gubner-Ungerleider, Sokolow-Lyon voltage, Sokolow-Lyon product, Cornell voltage, Cornell product, Romhilt-Estes 4-point and 5-point. Sensitivity, specificity, positive predictive valve (PPV), negative predictive value (NPV), and accuracy were calculated. Area under the receiver operator curve analysis (ROC-AUC) was performed.

## Results

**Figure 1:**
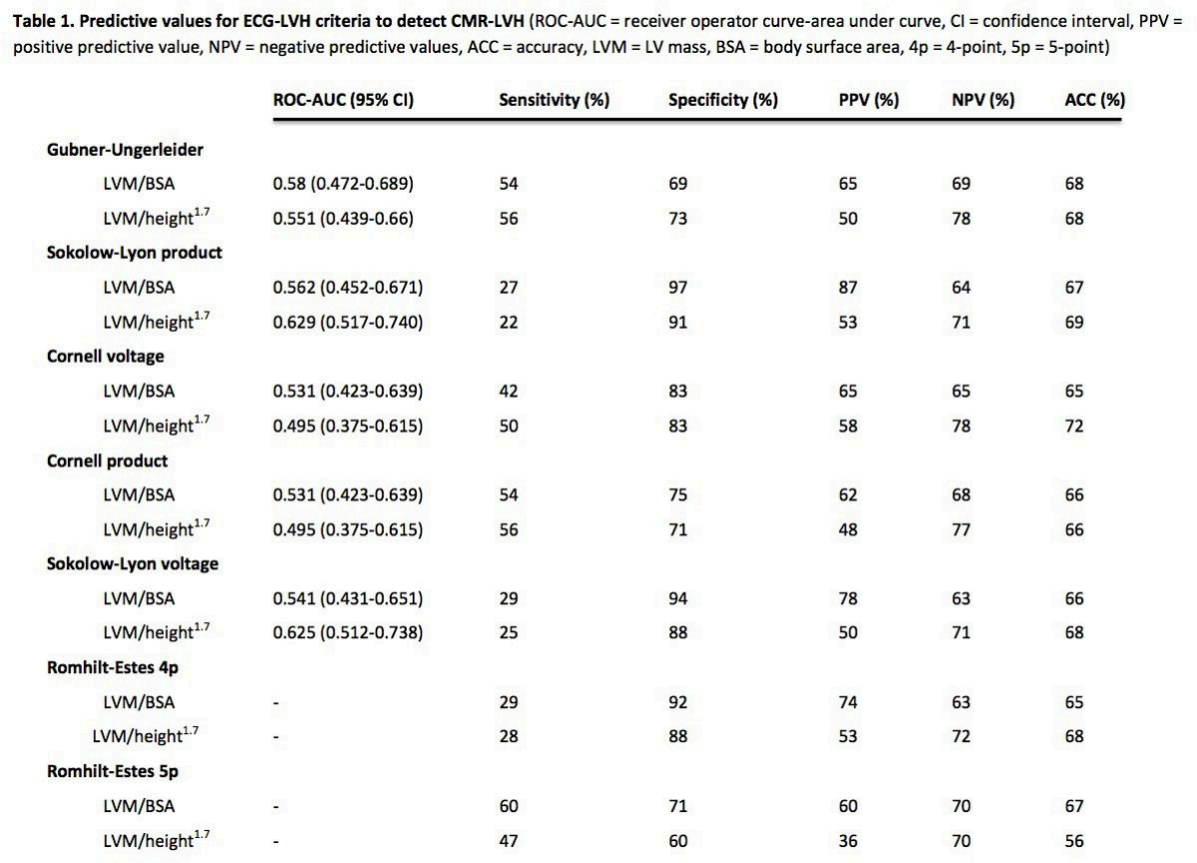
Predictive values for ECG-LVH criteria to detect CMR-LVH.

LVH was present in 43.2% by LVM/BSA and 32.4% by LVM/height^1.7^. There was no consistent trend in ROC-AUC values for detecting LVH as defined by LVM/height^1.7^ compared to LVM/BSA (Figure 1). The highest sensitivity (56%) was achieved by Gubner-Ugerleider and Cornell product) and the highest specificity (91%) by Sokolow-Lyon product for LVM/height^1.7^. Combining ECG criteria improved these sensitivities and specificities; if Gubner-Ugerleider or Cornell product were positive, sensitivity increased to 75% (accuracy 65%) and if both Sokolow-voltage product and Cornell voltage were negative, specificity increased to 99% (accuracy 71%).

**Figure 2:**
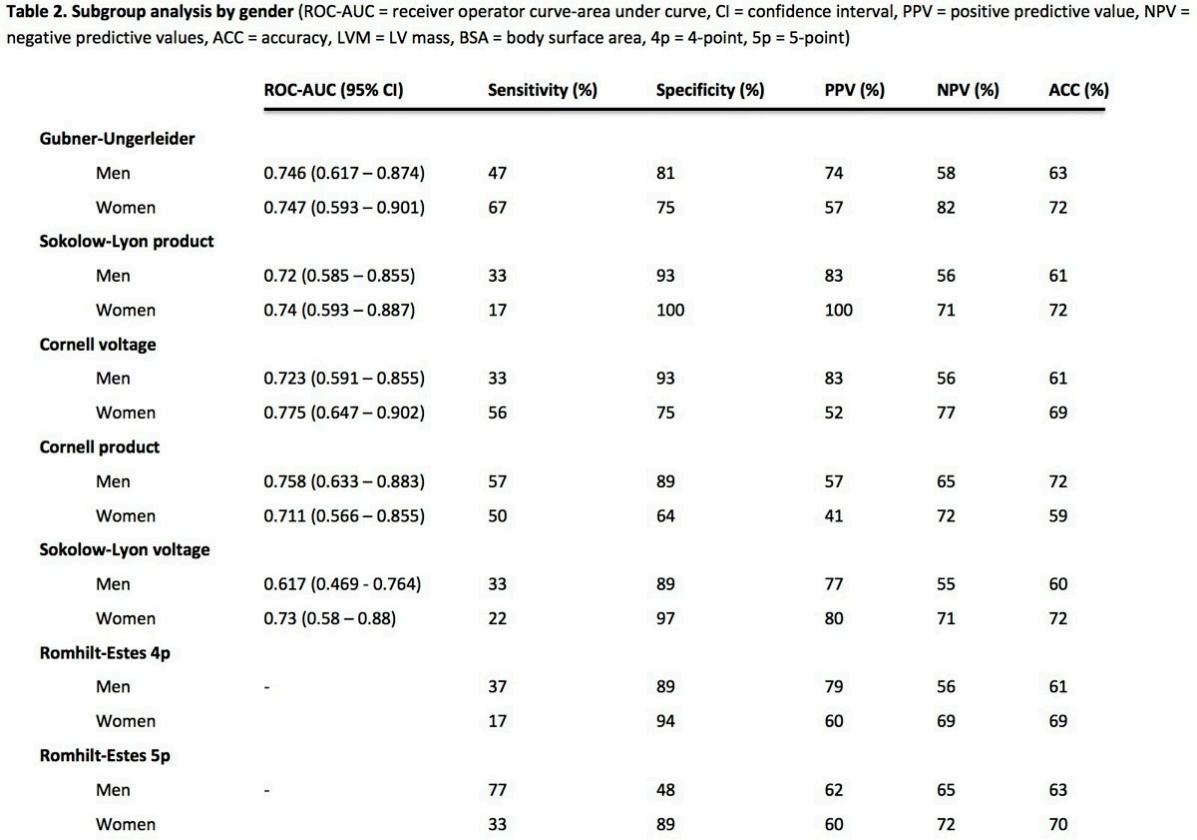
Subgroup analysis by gender.

Subgroup analysis by gender (Figure 2) revealed higher maximal sensitivity for men (77% by Romhilt-Estes 5-point) compared to women (67% by Gubner-Ugerleider) but lower maximal specificity for men (93% by Sokolow-Lyon product and Cornell voltage) compared to women (100% by Sokolow-Lyon product). There was a trend towards higher ROC-AUC values for women compared to men.

## Conclusions

Relative to CMR, standard 12-lead ECG criteria of LVH have a wide range of predictive values (but consistently greater specificity than sensitivity) in a tertiary hypertension clinic setting with high LVH prevalence. The poor diagnostic accuracy at detecting prognostically significant LVH highlights the ECG's limitations as a screenign tool for cardiac end-organ damage in hypertension. Combining ECG criteria and using different criteria for men and women improve the ECG's performance.

## Funding

NIHR Bristol Cardiovascular Biomedical Research Unit, Bristol Heart Institute.

JCLR: Clinical Society of Bath Postgraduate Research Bursary.

ECH: BHF grant IBSRF FS/11/1/28400.

